# Association of the blood levels of specific volatile organic compounds with nonfatal cardio-cerebrovascular events in US adults

**DOI:** 10.1186/s12889-024-18115-7

**Published:** 2024-02-26

**Authors:** Li Jing, Tiancong Chen, Zhiyong Yang, Weiwei Dong

**Affiliations:** 1 Department of Nursing, Shengjing Hospital of China Medical University, Shenyang, China; 2 Department of Neurosurgery, Shengjing Hospital of China Medical University, Shenyang, China; 3 Department of Cardiology, Shengjing Hospital of China Medical University, Shenyang, China

**Keywords:** VOCs, Myocardial infarction, Stroke, NHANES, Cross-sectional study

## Abstract

**Background:**

Cardio-cerebrovascular diseases constitute a major global public health burden. Volatile organic compounds (VOCs) exposure has become progressively severe, endangering human health and becoming one of the main concerns in environmental pollution. The associations of VOCs exposure with nonfatal cardio-cerebrovascular events have not been identified in observational study with a large sample size, so we aim to examine the association in US adult population.

**Methods:**

Adults aged > 18 years with complete data regarding selected blood levels of VOCs (including benzene, ethylbenzene, o-xylene, and m-/p-xylene) and nonfatal cardio-cerebrovascular events were included in the analysis (*n* = 3,968, National Health and Nutrition Examination Survey, NHANES, 2013–2018 survey cycle). Participants were classified into low- and high-exposure based on whether above selected VOCs low limit detect concentration or median value. Weighted multivariate logistic analyses and subgroup analyses were used to detect the association between selected VOCs exposure and nonfatal cardio-cerebrovascular events in US adults.

**Results:**

Weighted multivariate logistic analyses showed that the high-VOCs exposure group had an increased risk of nonfatal cardio-cerebrovascular events compared with the low-VOCs exposure group; the adjusted odds ratios (OR) and 95% confidence intervals (CI) of nonfatal cardio-cerebrovascular events for the high-VOCs exposure group were 1.41 (0.91, 2.19), 1.37 (0.96, 1.95), 1.32 (0.96, 1.82), and 1.17 (0.82, 1.67) for benzene, ethylbenzene, o-xylene, and m-/p-xylene, respectively, which was not significant assuming statistical significance at a 0.05 significance level (95% CI) for a two-tailed test. Lastly, we found high-VOCs exposure was associated with increased incidence of nonfatal cardio-cerebrovascular events in both daily smokers an non-daily smokers (*p-*interaction > 0.01), but the association was not statistically significant in non-daily smokers.

**Conclusions:**

This study found that VOCs (benzene, ethylbenzene, o-xylene, and m-/p-xylene) exposure was associated with increased incidence of nonfatal cardio-cerebrovascular events in US adults, and the results need to be confirmed by larger cohort studies.

**Supplementary Information:**

The online version contains supplementary material available at 10.1186/s12889-024-18115-7.

## Introduction

Globally, cardio-cerebrovascular diseases are the major causes of mortality and disability in the US despite increased preventive efforts [1]. Cardiovascular diseases account for 34% of all deaths in the US, with annual costs exceeding $500 billion [2]. Cardio-cerebrovascular disorders pose a great challenge to global health and economic burdens [3]. Further, cardio-cerebrovascular diseases remain an important threat to human health under conditions of increasing environmental pollution. It is important to identify environmental pollutant risk factors for cardio-cerebrovascular diseases as a complementary strategy for the prevention of cardio-cerebrovascular diseases [4–6].

The correlation between volatile organic compounds (VOCs) exposure and cardio-cerebrovascular disease has received more attention in recent years [7–9]. VOCs are considered to be risk factors for hypertension, endothelial injury, and cardiovascular events [10–12]. VOCs exposure is correlated with increasing risk of heart disease-related death, with a hazard ratio and 95% confidence interval of 1.39 (1.09–1.77) [13]. The four compounds in VOCs, including benzene, toluene, ethylbenzene, o-xylene, and m-/p-xylene, are collectively referred as BTEXs, and often analyzed together. Environmental and occupational pollution caused by VOCs is widespread from substances including tobacco smoke, paints, motor vehicle exhausts, furniture, and building materials [14, 15]. VOCs can cause non-cancerous health effects in humans even at low exposure levels [16]. The majority of studies on VOCs have focused on occupational exposures and had small sample size and specific exposure conditions. The effects of VOCs on cardio-cerebrovascular systems have rarely been investigated among the general population. The evidence on the association between VOCs exposure and cardio-cerebrovascular disease from well-designed, large-sample studies among the general population is still lacking. Hence, we performed a large cross-sectional investigation to identify the association between VOCs exposure and cardio-cerebrovascular diseases using surveillance data from National Health and Nutrition Examination Survey (NHANES) database. Higher VOCs exposure was hypothesized to be associated with a higher incidence of nonfatal cardio-cerebrovascular events.

## Materials and methods

### Study population

The study data were obtained from the NHANES database, which was established by the Center for Disease Control and Prevention (CDC) to assess the nutritional and health status of the US population. Because multi-stage stratified probability sampling was utilized in the NHANES study design, samples in NHANES are representative of the US population. Details of the NHANES survey and measurement methods are available online (https://wwwn.cdc.gov/nchs/nhanes/tutorials/default.aspx).All dataset used in this study was taken from publicly available dataset (https://wwwn.cdc.gov/nchs/nhanes/Default.aspx).The CDC provides free access to NHANES data via their official website. Our study included three NHANES survey cycles from 2013 to 2018, containing demographic, examination, laboratory, and questionnaire data. At first, 29,400 participants were enrolled in the study, and we excluded the individuals with missing data on stroke or myocardial infarction status (*n* = 19,941) and blood VOCs (benzene, ethylbenzene, o-xylene, and m-/p-xylene) concentration (*n* = 5491). A final number of 3,968 participants aged >18 years were enrolled in this study. The missing value in laboratory data was imputed via multiple imputation. The National Center for Health Statistics and Research Ethics Review Committee approved the use of human subjects for the NHANES study and written informed consent was provided by each participant.

### Exposure and outcome definitions

Participants visited a mobile examination clinic (MEC) where blood samples were collected. The vials were preserved under appropriate refrigerated (2–8 °C) conditions prior to shipment to the National Center for Environmental Health for analysis. Blood VOCs levels were measured via gas chromatography/mass spectrometry with selected-ion monitoring and isotope dilution. With this technique, blood VOCs levels in whole blood can be measured down to low parts per trillion. The NHANES Laboratory Procedures Manual contains detailed guidelines on sample collection and processing. The majority of 32 blood VOCs in NHANES had an extremely low detection rate. Benzene, ethylbenzene, o-xylene, and m-/p-xylene are with relatively higher detection rates approximately 30% and above in 32 blood VOCs and may affect the cardiovascular system, so we chose them for this study. The lowest limits of detectable blood VOCs concentrations and detection rates of selected VOCs in the blood are shown in Table 1. Blood VOCs concentrations below the limit of detection (LOD) were imputed by dividing the LOD by the square root of 2. To ensure reliable results, we also inputed blood VOCs concentrations below the LOD with 0 and the LOD value. There was no recognized cut point for the VOCs involved in this study, so we referred to the methodology of previous study and classified participants into low- and high-exposure based on whether above their LOD concentration or median value [17], as shown in Table 1.

We used the Medical Condition Questionnaire to determine outcomes. Our assessment method of defining a person with myocardial infarction or stroke was consistent with our previous studies [18, 19]. Previous epidemiological research employing NHANES data used these self-reported definitions of stroke and myocardial infarction and demonstrated that the self-reported assessment method was valid [20]. Participants self-reported myocardial infarction and/or stroke was used to define the outcome “nonfatal cardio-cerebrovascular event”.

### Covariates

Variables with a possible effect on the association of blood VOCs levels with nonfatal cardio-cerebrovascular events were gathered for this investigation based on the clinical experiences and existing literature. Demographic covariates included age, sex, race, poverty to income ratio (PIR), marital status, and educational level. The body mass index (BMI), systolic blood pressure (SBP), and diastolic blood pressure (DBP) were collected in the same way as our previous reports [18, 19]. Hypertension were defined as having a diagnosis of hypertension, taking antihypertensive medications, or having three consecutive SBP readings ≥ 140 mmHg or DBP readings ≥ 90 mmHg. Diabetes was defined as having a diagnosis of diabetes, taking hypoglycemic medications, glycated hemoglobin ≥ 6.5%, or fasting plasma glucose ≥ 126 mg/dL. Hyperlipidemia were defined as “yes” based on currently taking prescribed medication for hyperlipidemia or having been diagnosed with hyperlipidemia by a physician. Presence of sleep disorder was defined as “yes” based on diagnosis by a doctor of trouble sleeping. Physical activity (vigorous, moderate, no) in a typical week was defined as follows: Intensity of physical activity was classified as vigorous (activity that caused participants’ breathing or heart rate to increase significantly for at least 10 min continuously, such as construction work, or digging, lifting, carrying heavy loads), or moderate (activity that caused participants’ breathing or heart rate to increase slightly, such as carrying light loads or brisk walking for at least 10 min continuously) [18, 21]. The 2,5-dimethylfuran acted as a smoking biomarker with good sensitivity and specificity. The cutoff point for differentiating between current daily smokers and less than daily smokers (< 1 CPD), which includes nonsmokers, was set at a blood 2,5-dimethylfuran concentration of 0.014 ng/mL [22, 23]. We used this criteria to distinguish between daily smokers an non-daily smokers. Blood biochemical indices, including triglycerides (mg/dL), cholesterol (mg/dL), glucose (mg/dL), glycohemoglobin (%), and high-density lipoproteins (mg/dL) were tested based on the NHANES laboratory procedure manual(s) (http://www.cdc.gov/nchs/nhanes.htm).

### Statistical analyses

An appropriate NHANES sample weight was used in the analysis according to the CDC guidelines and the multistage cluster survey design. Continuous data are presented as mean ± standard error (SE), and categorical data are presented as percentages. When analyzing the intergroup differences, weighted linear regression was utilized to analyze measurement data and weighted chi-square test was used to analyze continuous data. Univariate and multivariate logistic analyses were utilized to calculate the odds ratio (OR) and 95% confidence interval (CI) to estimate the association between selected VOCs exposure and nonfatal cardio-cerebrovascular events. Weighted multivariate logistic regression was utilized in the multivariate analysis. Covariates that were known to be traditional or suspected risk factors for cardio-cerebrovascular or the estimates of the VOCs exposure on nonfatal cardio-cerebrovascular events changed by more than 10% in the multivariate logistic regression models. Stratified subgroup analysis was performed by smoking status, and the log-likelihood ratio test was utilized to detect the heterogeneity of the association in the presence or absence of daily smoking. R version 3.6.1 (https://www.r-project. org/) were used for statistical analyses. A *p*-value < 0.01 was considered statistically significant for all analyses.

## Results

### Baseline characteristics of the participants

The detection limits for the benzene, ethylbenzene, and o-xylene concentrations and m-/p-Xylene concentration were 0.024 and 0.034 ng/mL, respectively. The detection rates of benzene, ethylbenzene, o-xylene, and m-/p-xylene were 35%, 29%, 28%, and 65%, respectively. The median benzene level was 0.017 ng/mL (IQR, 0.017–0.043 ng/mL), median ethylbenzene level was 0.017 ng/mL (IQR, 0.017–0.030 ng/mL), median o-xylene level was 0.017 ng/mL (IQR, 0.017–0.027 ng/mL), and median m-/p-xylene level was 0.046 ng/mL (IQR, 0.024–0.096 ng/mL). The baseline characteristics of the selected VOCs are presented in Table 1. The incidence of nonfatal cardio-cerebrovascular events was high in the high-VOCs exposure group. The weighted incidences of nonfatal cardio-cerebrovascular events in high and low-VOCs exposure groups were 7.39% vs. 6.37% for benzene exposure, 8.05% vs. 6.15% for ethylbenzene exposure, 8.31% vs. 6.05% for o-xylene exposure, and 7.22% vs. 6.19% for m-/p-xylene exposure. The baseline characteristics of the weighted population are presented in Table 2.

**Table 1:** Baseline characteristics of selected blood VOCs in NHANES 2013–2018

VOCs	LOD(ng/mL)	Above LOD, *n*	Below LOD, *n*	Detect Rate(%)	Median (Q1-Q3) (ng/mL)	Low-exposure group, *n*	High-exposure group, *n*
Blood benzene	0.024	1380	2588	35	0.017 (0.017–0.043)	1380	2588
Blood Ethylbenzene	0.024	1166	2802	29	0.017 (0.017–0.030)	1166	2802
Blood o-Xylene	0.024	1126	2842	28	0.017 (0.017–0.027)	1126	2842
Blood m-/p-Xylene	0.034	2594	1374	65	0.046 (0.024–0.096)	1948	2020
BTEXs	0.106	1908	2060	48	0.103 (0.075–0.204)	1908	2060

VOCs, benzene, toluene, ethylbenzene and xylenes; LOD, Limit of detection, value below the LOD substituted as LOD/$\sqrt{2}$

VOCs exposure classified into high and low exposure group based on their low limit detect concentration or median value

BTEXs was the sum of benzene, Ethylbenzene, o-Xylene, and m-/p-Xylene

**Table 2:** Baseline characteristics of study participants in NHANES 2013–2018, weighted

	Blood Benzene		Blood Ethylbenzene		Blood o-Xylene		Blood m-/p-Xylene	
	Low-exposure	High-exposure	Low-exposure	High-exposure	Low-exposure	High-exposure	Low-exposure	High-exposure
Total (unweighted)	2588	1380	2802	1166	2842	1126	1948	2020
Total (weighted, US population estimate)	106,800,394	54,660,310	113,135,001	48,325,704	113,588,897	47,871,807	78,540,683	82,920,022
Age (years)	49.23±0.58	45.46±0.65	48.47±0.54	46.75±0.77	47.95±0.55	47.98±0.77	48.62±0.69	47.32±0.61
Sex, *n* (%)								
Male	46.71	52.13	45.60	55.45	45.83	54.98	43.01	53.79
Female	53.29	47.87	54.40	44.55	54.17	45.02	56.99	46.21
Race, *n* (%)								
Mexican American	8.86	8.72	10.14	5.70	10.38	5.09	9.41	8.25
Other races	17.31	14.32	17.15	14.31	17.39	13.71	17.31	15.34
Non-hispanic White	63.55	63.17	62.36	65.91	61.36	68.32	62.37	64.42
Non-hispanic Black	10.28	13.79	10.35	14.08	10.87	12.88	10.91	11.99
Educational level, *n* (%)								
<High school	11.00	17.77	11.15	18.32	11.69	17.10	10.99	15.48
High school	20.63	28.59	20.27	30.49	20.51	30.00	20.30	26.20
>High school	68.36	53.63	68.58	51.19	67.79	52.89	68.71	58.32
Marital status, *n* (%)								
Married/living with partner	66.13	57.75	64.34	60.84	63.81	62.06	63.58	63.02
Widowed/divorced/separated	16.80	20.85	17.32	20.16	17.52	19.70	17.52	18.78
Never married	17.07	21.41	18.34	19.01	18.67	18.24	18.89	18.20
PIR, *n* (%)								
< 1.2	16.47	26.35	17.25	25.82	18.51	22.90	17.73	21.79
≥ 1.2	83.53	73.65	82.75	74.18	81.49	77.10	82.27	78.21
BMI, *n* (%)								
< 25 kg/m^2^	24.10	29.50	25.28	27.45	25.24	27.56	25.51	26.33
25–30 kg/m^2^	33.67	31.84	31.99	35.54	32.25	34.96	31.48	34.54
≥ 30 kg/m^2^	42.22	38.66	42.73	37.00	42.51	37.48	43.01	39.13
Cholesterol (mg/dl)	190.86±1.36	191.50±1.83	190.89±1.26	191.51±1.76	190.31±1.26	192.89±1.87	189.22±1.58	192.83±1.27
Triglycerides (mg/dl)	148.24±3.69	152.37±4.82	149.32±3.85	150.40±4.09	149.03±3.81	151.08±4.84	146.46±4.59	152.65±3.68
HDL (mg/dl)	54.66±0.55	53.09±0.71	54.68±0.63	52.83±0.66	54.41±0.61	53.46±0.70	54.88±0.77	53.41±0.53
Serum cotinine (ng/mL)	10.93±1.82	133.20±8.58	14.73±2.35	140.33±9.56	20.19±2.41	128.58±9.85	12.27±2.25	90.27±5.67
Physicial activity, *n* (%)								
Vigorous	19.86	31.08	19.34	33.77	20.16	31.96	18.59	28.46
Moderate	25.73	22.88	25.66	22.67	25.87	22.14	26.26	23.34
No	54.41	46.04	55.00	43.56	53.97	45.90	55.14	48.20
Diabetes, *n* (%)								
Yes	17.15	12.72	16.91	12.70	16.44	13.78	17.23	14.15
No	82.85	87.28	83.09	87.30	83.56	86.22	82.77	85.85
Hypertension, *n* (%)								
Yes	41.07	38.14	39.73	40.91	39.42	41.65	39.43	40.70
No	58.93	61.86	60.27	59.09	60.58	58.35	60.57	59.30
Hyperlipidemia, *n* (%)								
Yes	38.39	30.43	36.69	33.36	36.42	33.97	36.47	34.97
No	61.61	69.57	63.31	66.64	63.58	66.03	63.53	65.03
Nonfatal cardiovascular event, *n* (%)								
Yes	6.37	7.39	6.15	8.05	6.05	8.31	6.19	7.22
No	93.63	92.61	93.85	91.95	93.95	91.69	93.81	92.78

Mean ± standard error (SE) for continuous data. Percentage (%) for categorical data

PIR, poverty to income ratio; BMI, body mass index; HDL, high density lipoprotein

### High VOCs exposure was associated with higher incidence of nonfatal cardio-cerebrovascular events

Weighted univariate logistic regression analysis revealed that the ORs with 95% CI of nonfatal cardio-cerebrovascular events for high exposure to the selected VOCs, benzene, ethylbenzene, o-xylene, m-/p-xylene, and their sum BTEXs were 1.17 (0.87, 1.58), 1.34 (1.00, 1.78), 1.41 (1.08, 1.84), 1.18 (0.86, 1.61), and 1.21 (0.88, 1.66), respectively, compared with those for the low exposure to these selected VOCs, as shown in Table 3.

High VOCs exposure was found to be positively associated with nonfatal cardiovascular events in the weighted multivariate logistic regression analysis, and the adjusted ORs and 95% CIs of nonfatal cardio-cerebrovascular events for high exposure to benzene, ethylbenzene, o-xylene,m-/p-xylene, and their sum BTEXs were 1.41 (0.91, 2.19), 1.37 (0.96, 1.95), 1.32 (0.96, 1.82), and 1.17 (0.82, 1.67), and 1.26(0.84, 1.89) respectively, after adjustment for age, sex, race, marital status, educational level, BMI, serum cotinine, hyperlipidemia, hypertension, and diabetes, as shown in Table 4. When imputed blood VOCs concentrations below LOD with 0 and the LOD value, we also found that high VOCs exposure tended to be positively associated with nonfatal cardiovascular events, as shown in Supplementary Table S1-S4. These results indicate that there was a positive association between selected VOCs exposure and nonfatal cardio-cerebrovascular events.

**Table 3:** Results of the univariate logistic regression analysis, weighted

	Statistic	OR (95%CI) *P*-value
Age (years)	47.95±0.46	1.07 (1.06, 1.08) < 0.0001
Sex, *n* (%)		
Male	48.55	Ref.
Female	51.45	0.83 (0.61, 1.12) 0.2351
Race, *n* (%)		
Mexican American	8.81	Ref.
Other races	16.30	0.72 (0.39, 1.34) 0.3048
Non-hispanic White	63.42	1.56 (1.04, 2.35) 0.0371
Non-hispanic Black	11.47	1.48 (0.99, 2.21) 0.0641
Educational level, *n* (%)		
<High school	13.30	Ref.
High school	23.33	0.86 (0.55, 1.34) 0.5144
>High school	63.37	0.56 (0.38, 0.83) 0.0059
Marital status, *n* (%)		
Married/living with partner	63.29	Ref.
Widowed/divorced/separated	18.17	1.80 (1.33, 2.45) 0.0005
Never married	18.54	0.41 (0.25, 0.66) 0.0007
PIR, *n* (%)		
< 1.2	19.82	Ref.
≥ 1.2	80.18	0.79 (0.58, 1.07) 0.1296
BMI, *n* (%)		
< 25 kg/m^2^	25.93	Ref.
25–30 kg/m^2^	33.05	1.36 (0.90, 2.05) 0.1468
≥ 30 kg/m^2^	41.02	1.72 (1.11, 2.66) 0.0187
Cholesterol (mg/dl)	191.08±1.20	0.99 (0.99, 1.00) 0.0151
Triglycerides (mg/dl)	149.64±3.21	1.00 (1.00, 1.00) 0.1423
HDL (mg/dl)	54.13±0.46	0.99 (0.97, 1.00) 0.1181
Serum cotinine (ng/mL)	52.34±2.69	1.03 (1.00, 1.06) 0.0396
Physicial activity, *n* (%)		
No	51.58	Ref.
Moderate	24.76	0.99 (0.69, 1.43) 0.9755
Vigorous	23.66	0.80 (0.53, 1.22) 0.3108
Diabetes, *n* (%)		
No	84.35	Ref.
Yes	15.65	4.48 (3.10, 6.47) < 0.0001
Hypertension, *n* (%)		
No	59.92	Ref.
Yes	40.08	3.69 (2.48, 5.49) < 0.0001
Hyperlipidemia, *n* (%)		
No	64.30	Ref.
Yes	35.70	5.14 (3.76, 7.03) < 0.0001
Blood benzene, *n* (%)		
Low-exposure group	66.15	Ref.
High-exposure group	33.85	1.17 (0.87, 1.58) 0.2968
Blood Ethylbenzene, *n* (%)		
Low-exposure group	70.07	Ref.
High-exposure group	29.93	1.34 (1.00, 1.78) 0.0549
Blood o-Xylene, *n* (%)		
Low-exposure group	70.35	Ref.
High-exposure group	29.65	1.41 (1.08, 1.84) 0.0159
Blood m-/p-Xylene, *n* (%)		
Low-exposure group	48.64	Ref.
High-exposure group	51.36	1.18 (0.86, 1.61) 0.3076
BTEXs, *n* (%)		
Low-exposure group	51.83	Ref.
High-exposure group	48.17	1.21 (0.88, 1.66) 0.2464

BTEXs was the sum of benzene, Ethylbenzene, o-Xylene, and m-/p-Xylene

PIR, poverty to income ratio; BMI, body mass index; HDL, high density lipoprotein; OR, odds ration; 95% CI, 95% confidence interval

**Table 4:** Results of the multivariate logistic regression analysis, weighted

	OR (95% CI) *p* value
Blood benzene	
Low-exposure group	Ref.
High-exposure group	1.41(0.91, 2.19) 0.1370
Blood Ethylbenzene	
Low-exposure group	Ref.
High-exposure group	1.37 (0.96, 1.95) 0.0924
Blood o-Xylene	
Low-exposure group	Ref.
High-exposure group	1.32 (0.96, 1.82) 0.0936
Blood m-/p-Xylene	
Low-exposure group	Ref.
High-exposure group	1.17 (0.82, 1.67) 0.3843
BTEXs	
Low-exposure group	Ref.
High-exposure group	1.26 (0.84, 1.89) 0.2651

Adjusted for age, sex, race, marital status, educational level, BMI, serum cotinine, hyperlipidemia, hypertension, and diabetes

BTEXs was the sum of benzene, Ethylbenzene, o-Xylene, and m-/p-Xylene

OR, odds ration; 95% CI, 95% confidence interval

### Subgroup analysis

We further conducted a subgroup analysis to determine the robustness and consistency of the association between selected VOCs exposure and nonfatal cardio-cerebrovascular events in the daily smokers an non-daily smokers when adjusted for age, sex, race, marital status, educational level, BMI, hyperlipidemia, hypertension, and diabetes. The association between selected VOCs exposure and non-fatal cardio-cerebrovascular events was not dependent on smoking status (all *p-*interaction > 0.01). High VOCs exposure was associated with an increased risk of nonfatal cardio-cerebrovascular events in both daily smokers an non-daily smokers, but the association was not statistically significant in non-daily smokers, as shown in Table 5.

**Table 5:** Results of subgroup analysis bases on smoke status

	OR (95%CI) *P*-value		*P*-interaction
	Smoke status (Yes)	Smoke status (No)	
Blood Benzene			0.3015
Low-exposure group	Ref.	Ref.	
High-exposure group	1.81 (1.10, 3.00) 0.0273	1.20 (0.65, 2.22) 0.5646	
Blood Ethylbenzene			0.3236
Low-exposure group	Ref.	Ref.	
High-exposure group	1.84 (1.29, 2.63) 0.0020	1.13 (0.49, 2.62) 0.7774	
Blood o-Xylene			0.3744
Low-exposure group	Ref.	Ref.	
High-exposure group	1.67 (1.17, 2.38) 0.0088	1.21 (0.67, 2.18) 0.5369	
Blood m-/p-Xylene			0.2723
Low-exposure group	Ref.	Ref.	
High-exposure group	1.50 (0.99, 2.26) 0.0645	1.10 (0.71, 1.72) 0.6637	
BTEXs			0.2341
Low-exposure group	Ref.	Ref.	
High-exposure group	1.62 (1.06, 2.48) 0.0340	1.18 (0.72, 1.93) 0.5125	

Adjusted for age, sex, race, marital status, educational level, BMI, hyperlipidemia, hypertension, and diabetes

BTEXs was the sum of benzene, Ethylbenzene, o-Xylene, and m-/p-Xylene

## Discussion

In the present study, we observed that high VOCs exposure was positively associated with nonfatal cardio-cerebrovascular events among adults in the US. In the subgroup analysis stratified with smoke status, we found the positive association between VOCs exposure and nonfatal cardio-cerebrovascular events was stable and robust.

Pollution caused by VOCs has been revealed to be correlated with oxidative stress, autonomic dysfunction, systemic inflammation, and blood coagulation, which are well-established risk factors for cardiovascular diseases [24, 25]. In animal models, mice exposed to volatile benzene exhibited a higher risk of developing cardiovascular diseases than those that breathed filtered air [26]. Benzene was found to be negatively correlated with many types of circulating angiogenic cells (CD45^dim^/CD146^+^/CD34^+^ cells) in human samples [11]. Abplanalp et al. found that mice inhalation of volatile benzene exhibited increasing plasma levels of low-density lipoproteins and decreasing plasma levels of circulating angiogenic cells (Flk-1+/Sca-1+) [26]. In other experiments, researchers also found that platelet macroparticles, percentage of CD4^+^ and CD8^+^ T-cells, and platelet-leukocyte aggregates were noticeably increased in mice that inhaled benzene [27]. Moreover, benzene exposure can aggravate heart failure by promoting neutrophil recruitment and endothelial activation [8]. Thus, VOCs exposure may aggravate cardio-cerebrovascular events by impairing the vascular endothelial function [28], and reduction in air VOCs concentrations was correlated with subsequent improvements in cardiovascular health [29, 30]. Meanwhile, exposure to VOCs also influenced other risk factors for cardio-cerebrovascular disease. Lee et al. revealed that benzene metabolites were associated with an increased risk of diabetes mellitus by utilizing data from the Korea National Environmental Health Survey [31]. VOCs exposure also found to be associated with metabolic syndrome [32]. However, the mechanisms responsible for thses association needs to be further investigated.

For the general public, the most significant source of exposure to VOCs is smoking. Consistent with this, this study found higher smoking rate was found in the high VOCs exposure group. In the subgroup analysis by presence or absence of daily smoking, We found that the positive association was was stable and robust in both daily smokers an non-daily smokers. The study of genetic risk factors for cerebrovascular disease revealed that single nucleotide polymorphisms (SNPs)-chromosome 8 loci with potential roles in vascular endothelial maintenance was independent of smoking [33]. The association between rs1333040 SNP and cerebrovascular disease was also found to be independent of smoking [34]. We hypothesized that smoking exposure may not alter genetic sensitivity to BTEXs and that exposure to BTEXs increased the risk of cardio-cerebrovascular disease to the similar extent in both smoking and nonsmoking participants. The positive association between high VOCs exposure and nonfatal cardiovascular or cerebrovascular events was stable and robust.

## Conclusion

VOCs exposure is a serious environmental pollution issue, there is increasing attention to VOCs toxic effect in human health. In this study, we observed positive association between benzene, ethylbenzene, o-xylene, m-/p-xylene and nonfatal cardio-cerebrovascular events, and the positive association was stable and robust in both daily smokers an non-daily smokers, but the association was not statistically significant in non-daily smokers. In the future, programs that engage in “source emissions environmental concentration-exposure human biological monitoring- health effects surveillance” are valuable and worth exploring for VOCs. Meanwhile, further research should investigate and identify the mechanistic hypothesis to explain the cardio-cerebrovascular hazard from VOCs exposure.

Our study also has several limitations. First, the cross-sectional design allowed us to conclude an association but not a causal inference. Second, unmeasured confounders may be a concern in our study. Although we controlled for a large number of confounding variables, the confounders, such as the use of other forms of smoked tobacco, second-hand smoke, marijuana were not included in this study, the association of BTEXs exposure and nonfatal cardio-cerebrovascular events may not be isolated fully accutate. Finally, VOCs are volatile and their levels in the blood may represent only recent exposure levels but not long-term exposure levels.

### Electronic supplementary material

Below is the link to the electronic supplementary material.

Supplementary Material 1

## Data Availability

All dataset used in this study was taken from NHANES publicly available dataset (https://wwwn.cdc.gov/nchs/nhanes/Default.aspx). The datasets used and/or analysed during the current study are available from the corresponding author on reasonable request.
