# Supplementary material for: Association of the blood levels of specific volatile organic compounds with nonfatal cardio-cerebrovascular events in US adults
Source: BMC Public Health. 2024 Feb 26;24:616. doi: 10.1186/s12889-024-18115-7 (PMC10898104; doi:10.1186/s12889-024-18115-7)
Supplement: Supplementary file 1 — Supplementary Material 1 [file 12889_2024_18115_MOESM1_ESM.docx]

**Table S1. Baseline characteristics of selected blood VOCs in NHANES 2013-2018 when the value below the LOD substituted as LOD.**

| VOCs | LOD(ng/mL) | Above LOD, n | Below LOD, n | Detect Rate(%) | Median (Q1-Q3) (ng/mL) | Low-exposure group, n | High-exposure group, n |
| --- | --- | --- | --- | --- | --- | --- | --- |
| Blood benzene | 0.024 | 1380 | 2588 | 35 | 0.024 (0.024 ,0.040) | 1380 | 2588 |
| Blood Ethylbenzene | 0.024 | 1166 | 2802 | 29 | 0.024 (0.024 ,0.031) | 1166 | 2802 |
| Blood o-Xylene | 0.024 | 1126 | 2842 | 28 | 0.024 (0.024 ,0.028) | 1126 | 2842 |
| Blood m-/p-Xylene | 0.034 | 2594 | 1374 | 65 | 0.047 (0.034 ,0.101) | 1990 | 1978 |

**Table S2. Results of the multivariate logistic regression analysis when the value below the LOD substituted as LOD, weighted.**

|  | OR (95% CI) p value |
| --- | --- |
| Blood benzene |  |
| Low-exposure group | Ref. |
| High-exposure group | 1.73 (1.16, 2.59) 0.0123 |
| Blood Ethylbenzene |  |
| Low-exposure group | Ref. |
| High-exposure group | 1.69 (1.19, 2.41) 0.0068 |
| Blood o-Xylene |  |
| Low-exposure group | Ref. |
| High-exposure group | 1.60 (1.16, 2.20) 0.0074 |
| Blood m-/p-Xylene |  |
| Low-exposure group | Ref. |
| High-exposure group | 1.43 (1.00, 2.05) 0.0523 |

Adjusted for age, sex, race, marital status, educational level, BMI, hyperlipidemia, hypertension, and diabetes.

**Table S3. Baseline characteristics of selected blood VOCs in NHANES 2013-2018 when the value below the LOD substituted as 0.**

| VOCs | LOD(ng/mL) | Above LOD, n | Below LOD, n | Detect Rate(%) | Median (Q1-Q3) (ng/mL) | Low-exposure group, n | High-exposure group, n |
| --- | --- | --- | --- | --- | --- | --- | --- |
| Blood benzene | 0.024 | 1380 | 2588 | 35 | 0.000 (0.000 ,0.040) | 1380 | 2588 |
| Blood Ethylbenzene | 0.024 | 1166 | 2802 | 29 | 0.000 (0.000 ,0.031) | 1166 | 2802 |
| Blood o-Xylene | 0.024 | 1126 | 2842 | 28 | 0.000 (0.000 ,0.028) | 1126 | 2842 |
| Blood m-/p-Xylene | 0.034 | 2594 | 1374 | 65 | 0.047 (0.000 ,0.101) | 1990 | 1978 |

**Table S4. Results of the multivariate logistic regression analysis when the value below the LOD substituted as 0, weighted.**

|  | OR (95% CI) p value |
| --- | --- |
| Blood benzene |  |
| Low-exposure group | Ref. |
| High-exposure group | 1.73 (1.16, 2.59) 0.0123 |
| Blood Ethylbenzene |  |
| Low-exposure group | Ref. |
| High-exposure group | 1.69 (1.19, 2.41) 0.0068 |
| Blood o-Xylene |  |
| Low-exposure group | Ref. |
| High-exposure group | 1.60 (1.16, 2.20) 0.0074 |
| Blood m-/p-Xylene |  |
| Low-exposure group | Ref. |
| High-exposure group | 1.43 (1.00, 2.05) 0.0523 |

Adjusted for age, sex, race, marital status, educational level, BMI, hyperlipidemia, hypertension, and diabetes.
